# Beyond Ophthalmoplegia: Wernicke Encephalopathy with Anisocoria

**DOI:** 10.3390/reports9040341

**Published:** 2026-10-09

**Authors:** Yoshihiro Ito, Koji Hayashi, Haruka Kurihara, Yuka Nakaya, Mamiko Sato

**Affiliations:** 1 Department of Rehabilitation Medicine, Fukui General Hospital, Fukui 910-8561, Japansatomoko@f-gh.jp (M.S.); 2 Graduate School of Health Science, Fukui Health Science University, Fukui 910-3113, Japan

**Keywords:** Wernicke encephalopathy, thiamine deficiency, anisocoria, alcoholism, autonomic nervous system diseases, diagnosis, differential

## Abstract

A 69-year-old man with a history of heavy alcohol consumption and poor nutrition presented with disorientation and gait instability. The cognitive assessment was severely affected, with the Hasegawa Dementia Scale-Revised score was 4. Neurological examination revealed anisocoria (right 4.0 mm, left 2.0 mm) with sluggish light reflexes, gaze-evoked horizontal nystagmus, and vertical gaze restrictions. He exhibited disorientation and limb ataxia; deep tendon reflexes were normal. Brain MRI showed bilateral thalamic hyperintensities on diffusion-weighted imaging and periaqueductal high signal on T2 FLAIR. Laboratory testing confirmed a severely reduced serum thiamine level of 10.5 ng/mL, establishing Wernicke encephalopathy (WE) based on Caine’s criteria. Immediately upon admission (Day 1), high-dose thiamine therapy was initiated at 700 mg/day (300 mg/day intravenous drip plus 400 mg/day oral) for 7 days, followed by 400 mg/day oral. Despite prompt, high-dose thiamine administration, the 2.0 mm anisocoria persisted consistently throughout the 19-day hospitalization, whereas cognitive function and ataxia partially improved. The 2.0 mm interocular difference confirmed pathologic anisocoria, indicating asymmetric autonomic disruption involving descending sympathetic pathways in the periaqueductal gray matter. While neuro-ophthalmic signs in WE are typically reversible, this case demonstrates that autonomic deficits may persist despite comprehensive thiamine therapy, highlighting WE as a critical metabolic mimic of life-threatening structural brainstem disorders.

Brain MRI is considered the most valuable diagnostic tool to support a clinical diagnosis of Wernicke encephalopathy (WE) because of its high specificity (approximately 93%), although its sensitivity is relatively low (approximately 53–65%) [1,2]. Importantly, a normal brain MRI cannot exclude the diagnosis of WE [2]. The characteristic MRI features of WE are defined by bilateral, symmetric signal abnormalities typically located in the medial thalami, mammillary bodies, periaqueductal gray matter, and tectal plate, which characteristically manifest as hyperintensities on T2-weighted and FLAIR sequences and hypointensities on T1-weighted images [2,3,4]. Furthermore, diffusion-weighted imaging (DWI) is highly sensitive for early-stage detection, often revealing hyperintense signals that reflect either cytotoxic edema with decreased apparent diffusion coefficient (ADC) values or vasogenic edema with normal-to-increased ADC values [2,4], while post-contrast T1-weighted sequences frequently demonstrate mammillary body enhancement indicative of blood-brain barrier disruption [3,4]. Although contrast-enhanced MRI was not performed, the findings on non-contrast MRI in this case were consistent with typical WE (Figure 1).

**Figure 1:**
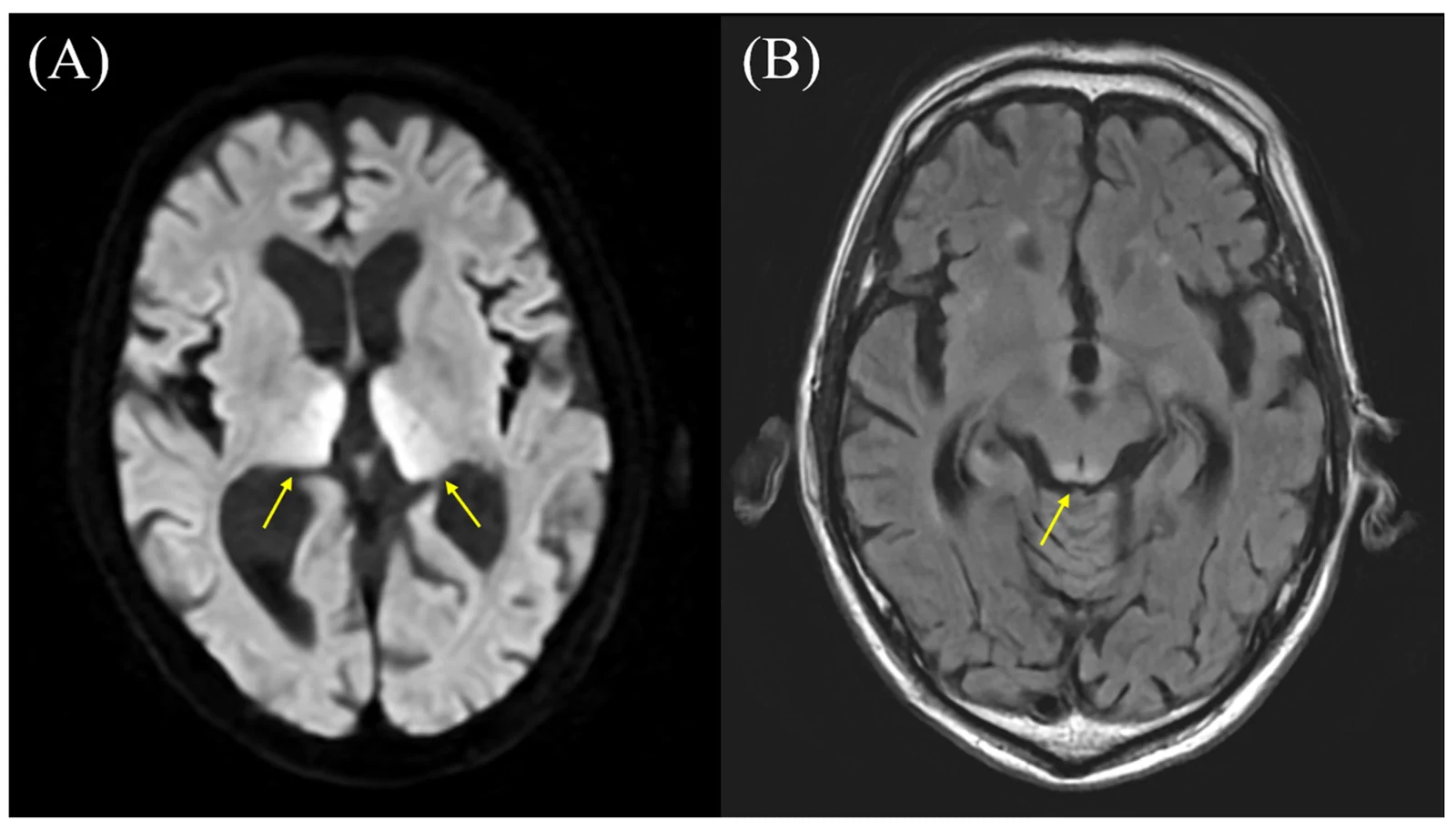
Brain magnetic resonance imaging (MRI) findings. (**A**) Diffusion-weighted imaging (DWI) showing hyperintensities in the bilateral thalami (arrows). (**B**) T2-fluid-attenuated inversion recovery (FLAIR) imaging showing hyperintensity in the dorsal region surrounding the cerebral aqueduct (arrow). The patient is a 69-year-old man with a history of chronic heavy alcohol consumption and poor diet. He presented with a multi-day history of progressive disorientation and gait instability. On emergency admission (Day 1), baseline laboratory testing confirmed a severely decreased pre-treatment serum thiamine concentration of 10.5 ng/mL (reference range: 24.0–66.0 ng/mL). The diagnosis of Wernicke encephalopathy (WE) was formally established using Caine’s criteria [5], as the patient fulfilled all four diagnostic features: (1) dietary deficiency/alcoholism, (2) oculomotor abnormalities (gaze-evoked horizontal nystagmus and vertical gaze restriction), (3) cerebellar limb/gait ataxia, and (4) altered mental status (Hasegawa Dementia Scale-Revised score: 4). Immediately upon diagnosis on Day 1, high-dose thiamine replacement therapy was initiated. The regimen consisted of 700 mg/day (300 mg/day intravenous drip combined with 400 mg/day oral supplementation) for the first 7 days (Days 1–7), followed by continuous oral thiamine supplementation at 400 mg/day from Day 8 onward throughout the 19-day acute care hospitalization.

**Figure 2:**
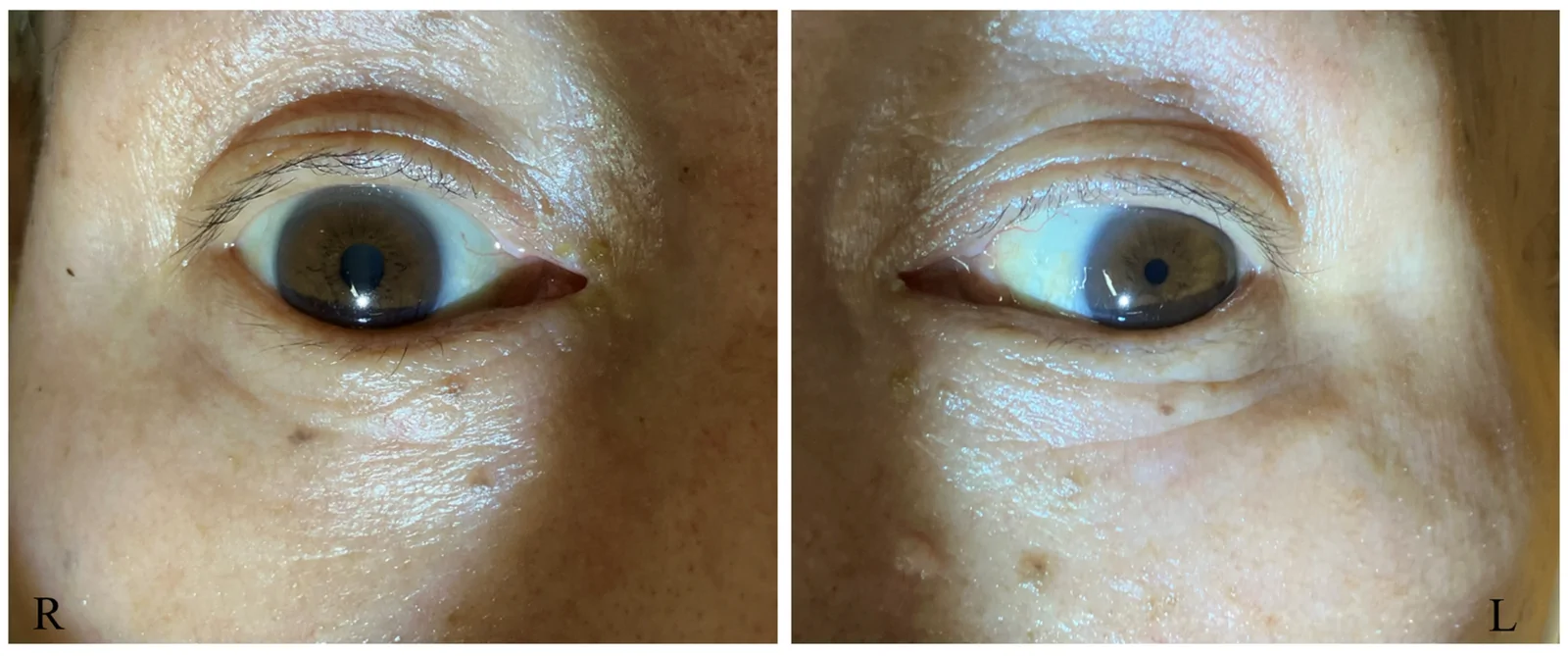
Photograph of the patient’s pupils. The (**left**) pupil is constricted, demonstrating marked anisocoria ((**right**) pupil: 4.0 mm, (**left**) pupil: 2.0 mm). Additionally, bilateral sluggish light reflexes were noted. In this case, the patient developed cognitive decline consistent with Wernicke–Korsakoff syndrome. Information regarding prior eye surgery, baseline pupillary asymmetry, or past ophthalmological visits was unavailable due to vague responses. However, alternative causes of anisocoria were clinically and radiologically excluded: third cranial nerve palsy was ruled out by the complete absence of ptosis or isolated extraocular muscle weakness; Horner’s syndrome was unlikely given the lack of ptosis or anhidrosis; acute brainstem infarction, hemorrhage, or mass lesions were excluded on brain MRI; and there was no history of ocular trauma or exposure to systemic/topical autonomic medications. Furthermore, physiologic anisocoria is usually small—commonly less than 0.5 mm—whereas anisocoria of 1 mm or greater is uncommon in healthy individuals [6]. In a quantitative pupillometry study involving healthy volunteers, only 1.5% demonstrated a resting interocular difference of 1 mm or more [6]. Given that the observed asymmetry in our patient was 2.0 mm—and considering that a difference of 1.0 mm or more is frequently used in neurocritical care research as the operational definition of “new-onset anisocoria” [7]—this finding was interpreted as representing a true pathologic autonomic asymmetry rather than longstanding physiologic variation.

In WE, pupillary findings are significant neuro-ophthalmic manifestations, although they are less frequently observed than the classic triad of altered mental status, nystagmus, and ataxia [3]. Pupillary abnormalities reported to date in association with WE include diminished light reflex, miosis, pinpoint pupils, and anisocoria [2,3,8,9,10]. Pathophysiologically, the affected pupil’s size is hypothesized to result from damage to the descending sympathetic pathways that originate in the hypothalamus and pass through the brainstem (including the periaqueductal gray and periventricular tissues), areas characteristically affected by thiamine deficiency [9,10]. Additionally, the diminished light reflex is thought to be related to metabolic lesions in the midbrain aqueduct region, specifically involving the Edinger-Westphal nucleus and oculomotor complex [3,9].

In the present case in Figure 2, the co-occurrence of anisocoria and the periaqueductal FLAIR hyperintensity suggests that the descending sympathetic fibers were asymmetrically involved. This finding highlights two vital clinical insights. First, while WE is typically characterized by bilateral symmetric lesions, the presence of anisocoria demonstrates that autonomic fibers might be asymmetrically disrupted, revealing a more complex and heterogeneous pathophysiology than is commonly recognized. Second, because anisocoria is a hallmark of high-urgency conditions such as acute brainstem infarction or uncal herniation, clinicians must maintain a high index of suspicion for WE as a critical metabolic “mimic” of these life-threatening events. Notably, high-dose thiamine therapy was initiated immediately on Day 1 (700 mg/day [300 mg/day IV drip combined with 400 mg/day oral] for 7 days, followed by 400 mg/day oral). While most neuro-ophthalmic manifestations of WE typically resolve rapidly with treatment [4,11], the complete lack of pupillary improvement throughout the 19-day management period demonstrates that autonomic fiber injury in WE can be structural and poorly reversible despite prompt, comprehensive thiamine rescue.

This case report has several limitations. Due to the patient’s severe cognitive impairment (Hasegawa Dementia Scale-Revised score: 4) and acute clinical constraints, formal pharmacological pupillary testing (e.g., sympathomimetic or mydriatic eye-drop challenges), accommodation testing, and quantitative pupillometry under light/dark conditions were not performed. Consequently, whether the defect corresponds predominantly to a miotic or mydriatic pupil could not be functionally verified, limiting definitive anatomical localization—specifically distinguishing whether the anisocoria resulted from asymmetric descending sympathetic tract damage, parasympathetic involvement, or pretectal lesions. Furthermore, without pathological verification of the brainstem, this neuroanatomical tract disruption remains a plausible hypothesis rather than a proven certainty. Nevertheless, the combination of acute 2.0 mm anisocoria (exceeding the 1.0 mm operational threshold for new-onset pathological anisocoria), low pre-treatment serum thiamine levels (10.5 ng/mL), and characteristic periaqueductal/tectal MRI hyperintensities strongly implicates thiamine deficiency as the underlying etiology.

## Data Availability

The original contributions presented in this study are included in the article. Further inquiries can be directed to the corresponding author.
